# Microsatellite analysis revealed the genetic diversity and population structure of 18 native black goat breeds in China

**DOI:** 10.5713/ab.25.0224

**Published:** 2025-06-24

**Authors:** Tao Zhang, Jiaxue Guo, Ge Qin, Guangxin E, Deli Huang, Yan Zeng, Yongju Zhao, Zhongquan Zhao, Yongfu Huang, Yanguo Han

**Affiliations:** 1 Southwest University, Chongqing, China; 2 College of Animal Science and Technology, Southwest University, Chongqing Engineering Research Centre for Herbivores Resource Protection and Utilization, Chongqing Key Laboratory of Forage & Herbivore, Chongqing, China; 3 Chongqing Tengda Animal Husbandry Co., Ltd., Chongqing, China

**Keywords:** Black Goats, Genetic Diversity, Microsatellites, Population Structure

## Abstract

**Objective:**

In China and Southeast Asia, black goats command higher selling prices. However, the blind breeding practices carried out by farmers pose a threat to the original genetic diversity of the population. Therefore, the objective of this study is to conduct a systematic detection of the genetic diversity of native black goat breeds, aiming to provide a reference for the protection and improvement of these valuable native black goat breeds.

**Methods:**

Genetic diversity and population structure of 18 black goat breeds were estimated by utilizing 16 microsatellite markers. Subsequently, data analysis was carried out with the assistance of software like Phylip, Fstat, Arlequin, Structure. For the purpose of visualization, ITOL and Structure Selector were used to present the results in a visual manner.

**Results:**

The mean number of alleles per population ranged from 4.75 to 9.56, with an average of 6.38. The observed heterozygosity of each breed ranged from 0.46 to 0.68, all of which were lower than the expected heterozygosity. The inbreeding coefficient (*F*_IS_) of the 18 breeds ranged from −0.003 to 0.376. Among them, the *F*_IS_ values of Meigu goat (MG), Yimeng black goat, Yunling goat, Guizhou black goat and Ziwuling black goat were significantly higher than those under random rearrangement (p<0.05). All pairwise Fixation index between the Chinese black goat populations reached a significant level (p<0.05). Finally, the results of Bayesian model-based clustering and a neighbor-joining tree based on Nei’s genetic distance showed these eighteen breeds can be further classified into seven genetic clusters.

**Conclusion:**

All breeds showed high genetic diversity. MG had excessive inbreeding, and CZ and LZ were at risk of losing original genetic traits. Similar geographical and climatic conditions might lead to similar genetic materials in different breeds.

## INTRODUCTION

In recent years, chevon, boasting its attributes of high protein and low fat, along with an abundance of unsaturated fatty acids (linoleic acid and linolenic acid), is becoming increasingly popular among people, and its production has been increasing year by year [1]. As a livestock breed with extremely strong adaptability, goats are widely distributed around the world, especially in Asia and Africa [2]. In East Asia and Southeast Asia, due to the influence of traditional customs, local communities show a distinct preference for black. Correspondingly, black goats are more popular and command higher prices [3, 4]. China has a rich diversity of black goat breeds. According to the statistics of the Chinese National Germplasm Center of Domestic Animals, there are approximately 45 breeds [5]. In order to obtain higher profits, farmers raising black goats will also carry out certain breeding and hybridization programs. However, due to the lack of scientific guidance, these programs are often carried out blindly in many cases. To date, no systematic research has been conducted to analyze black goat breeds genetic diversity and population structure.

Microsatellite markers have been widely used as a method for assessing genetic diversity in the evaluation of the genetic diversity of goats. For example, dairy goats [6, 7], cashmere goats [8, 9] and meat goats [10]. In addition, some researchers have utilized microsatellite markers to study the population structure and phylogenetic relationships of goats in different regions [11 – 13]. In this study, 16 microsatellite markers were used to evaluate the genetic diversity and population structure of 18 black goat breeds in China. The study was expected to provide the scientific conservation and breeding plans for native black goat breeds.

## MATERIALS AND METHODS

### Experimental animals and DNA extraction

A total of 661 black goats from eighteen breeds/types spanning the entire distribution range of Chinese black goats were analysed. Samples for each breed were obtained from conservation farms in their native regions. From the population of healthy adult goats with breed-typical characteristics across different pedigrees, 10% were randomly selected as experimental subjects. All experimental procedures were approved by the Animal Care and Use Committee of Southwest University (No. IACUC-20240920-07). The specific number of samples for each breed and their geographical locations are shown in Table 1. Samples were obtained by collecting blood from the jugular vein. The genomic DNA was extracted following the standard phenol: chloroform protocol [14]. The quality of the DNA was evaluated by means of a 0.8% agarose gel, and the extracted DNA was quantified using a DTX microplate reader (Beckman Coulter).

### Polymerase chain reaction amplification and sequencing

All goats were genotyped using 16 microsatellite markers (Table 2) recommended by the Food and Agriculture Organization of the United Nations [15]. The 10 μL polymerase chain reaction (PCR) reaction system includes 2×Taq PCR Master Mix, 5μL; template DNA, 1μL; primer F and primer R (10 pmol/μL), each is 0.5μL; ddH_2_O, 3 μL. The reaction program is as follows: pre-denaturation 95°C for 5 min; denaturation 95°C for 30 s, annealing at 55°C or 58°C (the annealing temperatures vary for different primers.) for 30 s, 72°C extension for 30 s, 10 cycles (annealing temperature reduced 1°C per cycle); denaturation 95°C for 30 s, 52°C annealing for 30 s, 72°C extension for 30 s, 25 cycles; extension at 72°C for 20 min. Sequencing was carried out using the ABI 3730xl (Applied Bio Systems). After the raw data in the .fsa format were exported from the ABI 3730xl, they were imported into the Gene Marker (Promega) analysis software to obtain the original genotypic data.

### Statistical analysis

The original data were analyzed using the Microsatellites toolkit [16] to obtain the number of alleles (NA), the mean number of alleles (MNA), the effective number of alleles (NEA), the number of private alleles (NPA), the expected heterozygosity (*H*_E_), the observed heterozygosity (*H*_O_), and the polymorphic information content (PIC). The allelic richness (AR) of each population was calculated using the Fstat v1.2. [17] Arlequin 3.5. [18] was used to calculate whether the microsatellite loci deviated from the Hardy-Weinberg equilibrium (HWE), the genetic differentiation index (*F*_ST_), and the inbreeding coefficient (*F*_IS_). The Phylip software package [19] was utilized to construct a Neighbor-Joining (NJ) tree based on the DA genetic distance. Subsequently, the resulting tree was visualized using the online software Itol [20]. To infer the genetic structure of the breeds, the software Structure v2.3.4. [21] was applied with a Bayesian clustering approach 15. Finally, the optimal K value was determined using the online software Structure Selector [22], and a corresponding clustering diagram was generated to illustrate the genetic relationships among the breeds.

## RESULTS

### The polymorphism of microsatellite loci

At the 16 selected loci, the average NA was 20.5 (Table 3). Among them, the loci SRCRSP23 and TCRVB6 had the largest NA, which was 30, while there were only 7 alleles at the MAF209 locus, which was the least among the 16 loci. The average NEA at the loci was 3.30 (ranging from 1.62 [MAF209] to 4.96 [SRCRSP8]). The average *H*_O_ and *H*_E_ were 0.59 and 0.63 respectively (ranging from 0.22 [SRCRSP7] to 0.74 [SRCRSP8] for *H*_O_ and from 0.36 [MAF209] to 0.80 [SRCRSP8] for *H*_E_), and the average PIC was 0.59 (ranging from 0.29 [MAF209] to 0.76 [SRCRSP8]). Except for a few loci, the PIC of the remaining loci was higher than 0.5.

### The genetic diversity of the breeds

By using 16 microsatellite markers to evaluate the genetic diversity of black goats from different breeds, we found that MG had the largest total NA, which was 153, while JC had the least NA (76) (Table 4). Correspondingly, MG had the highest MNA and AR, which were 9.56 (4.19) and 7.26 (2.45) respectively; JC had the lowest MNA and AR, which were 4.75 (1.53) and 4.02 (1.17) respectively. However, in terms of the indicator of the NEA at the loci, LW had the highest average NEA, which was 4.18 (1.70), while FQ had the lowest average NEA (2.65 [0.90]). The total NA of these two were neither the highest nor the lowest. In this study, the range of the *H*_O_ of each population was 0.46 to 0.68, and the range of the *H*_E_ was 0.59 to 0.74. The *H*_O_ of all populations was lower than the *H*_E_. Thirteen loci in MG deviated from the HWE. There were no loci deviating from the HWE in DZ and BY. The average number of loci deviating from the HWE for all breeds was 3.39.

### Intra-population inbreeding and inter-population genetic differentiation

**Figure 1:**
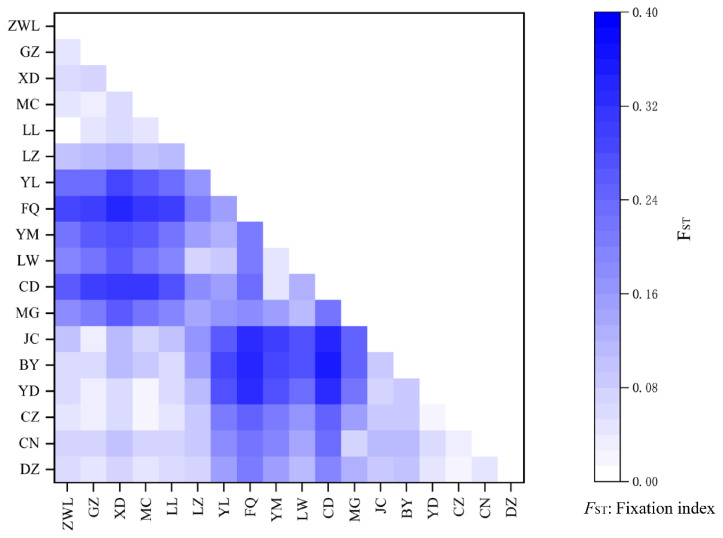
Population average pairwise differences of 18 Chinese black goat breeds. ZWL, Ziwuling; GZ, Guizhou; XD, Xiangdong; MC, Macheng; LL, Lvliang; LZ, Leizhou; YL, Yunling; FQ, Fengqing; YM, Yimeng; LW, Laiwu; CD, Chengde; MG, Meigu; JC, Jianchang; BY, Baiyu; YD, Yudong; CZ, Chuanzhong; CN, Chuannan; DZ, Dazu. (These are all breed names of Chinese native black goats.)

In order to evaluate the degree of inbreeding within populations and the genetic differentiation between populations, *F*-statistics were calculated. The *F*_IS_ values of the 18 populations ranged from −0.003 to 0.376, showing a relatively large span. Among them, the *F*_IS_ values of MG, YM, YL, GZ, and ZWL were significantly higher than those under random rearrangement (p<0.05). The calculated values of *F*_ST_ are shown in Table 5 and Figure 1 and their significance is corrected using the Bonferroni method [23]. According to the definition by Sewall Wright [24], *F*_ST_ is classified into four levels: low (*F*_ST_<0.05), moderate (0.05<*F*_ST_<0.15), high (0.15<*F*_ST_<0.25), and extremely high (*F*_ST_>0.25). As shown in Figure 1, The *F*_ST_ values of YL, FQ, YM, LW, CD and MG were greater than 0.15 or 0.25 when compared with those of other breeds. This indicated that there was a high or extremely high level of genetic differentiation between them and other breeds.

### Phylogenetic relationship and population structure of 18 populations

**Figure 2:**
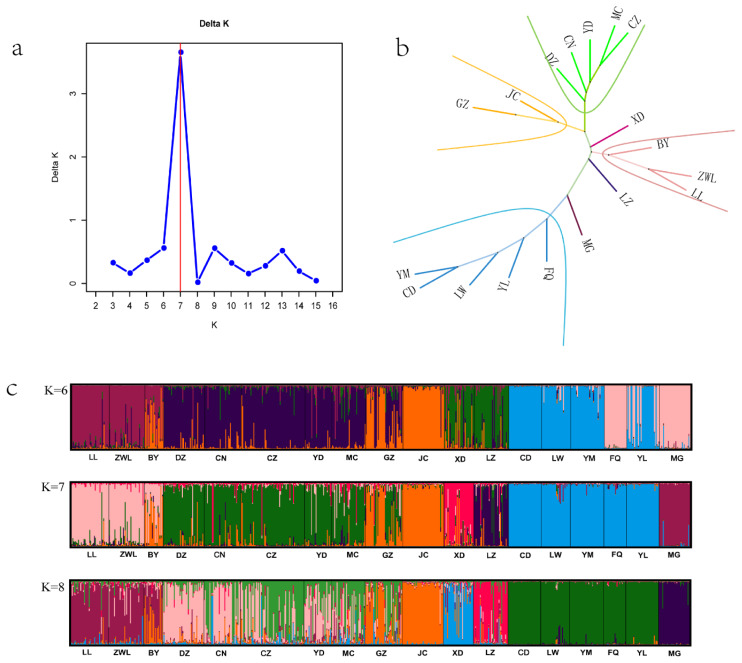
Phylogenetic tree and population structure clustering diagram of 18 black goat breeds. (A) Diagram of \Delta K values exported by Structure Selector. (B) Phylogenetic tree diagram of 18 goat breeds. (C) Clustering diagrams at K values of 6, 7, and 8. LL, Lvliang; ZWL, Ziwuling; BY, Baiyu; DZ, Dazu; CN, Chuannan; CZ, Chuanzhong; YD, Yudong; MC, Macheng; GZ, Guizhou; JC, Jianchang; XD, Xiangdong; LZ, Leizhou; CD, Chengde; LW, Laiwu; YM, Yimeng; FQ, Fengqing; YL, Yunling; MG, Meigu. (These are all breed names of Chinese native black goats.)

The clustering diagram can be used to analyze the population structure and estimate the number of subgroups within the population. According to Figure 2 A, when K = 7, the value of ΔK [25] was the largest, indicating that the optimal number of subgroups was 7. Figure 2 B was a phylogenetic tree of 18 populations. According to Figure 2 C, the clustering could be carried out as shown in the figure. JC and GZ were classified into one cluster; DZ, CN, YD, MC and CZ were classified into one cluster; BY, ZWL and LL were classified into one cluster; YM, CD, LW, YL and FQ were classified into one cluster; in addition, XD, LZ and MG were respectively classified into one cluster. Figure 2 C was a structural clustering diagram of 18 goat populations under the division of 6, 7 and 8 subgroups. It was known that the division into 7 subgroups was the optimal grouping method. Different colors on the clustering diagram indicated belonging to different subgroups. From the colors, it could be seen that in addition to the main color (the color with the largest proportion in the clustering diagram of a specific breed, such as the green color for DZ in Figure 2 C), there were more or less several other colors (in the clustering diagram of a specific breed, minor color components are observed, such as the red, purple, and yellow hues for DZ in Figure 2 C) among the 18 populations, indicating that there were hybridization situations with other breeds.

## DISCUSSION

In this study, we evaluated the genetic diversity and population structure of 18 native black goat breeds in China using 16 microsatellite loci. The results showed that the NA, heterozygosity, and PIC of the 16 loci all exhibited relatively high levels. This indicated that the selected loci can effectively assess the diversity of the populations. The genetic variability quantified for each goat population is presented in Table 4. Vellnow et al [26] pointed out that the closer the NA was to the NEA, the more evenly the alleles were distributed within the breed. In this study, there were differences between the NEA and the NA for all breeds, which might be caused by artificial breeding [11]. Kreling et al [27] pointed out that the higher the breed-specific polymorphism of a breed, the lower gene exchange there was between it and other breeds. The MG breed had the highest NPA. Combining with the research of Kreling et al, it showed that the MG breed had less gene exchange with other breeds. In the study of Wei et al [28] on the genetic structure of native Chinese goat breeds, the genetic diversity of some of the goat breeds in this study (YL, JC, LZ, YM, LL, XD, FQ) was evaluated. The *H*_O_ of YL (0.55), JC (0.57), LZ (0.53), YM (0.56), LL (0.65), and XD (0.55) were highly similar to the study by Wei et al. Nevertheless, there was a significant deviation between the *H*_O_ of FQ in this study (0.57) and that in Wei’s study (0.52). Apart from the sampling error, it might also be related to introduction (introduction of new goat breeds) and cross – breeding [29]. Compared with relevant studies on goats in Europe and the Middle East [30], the average *H*_O_ (0.59) and *H*_E_ (0.63) of Chinese goats in this study were both lower than those of European goats (*H*_O_ = 0.62, *H*_E_ = 0.69). This suggested that the degree of genetic variation in Chinese goats was lower than that in European goat breeds, which was consistent with the results of previous studies on the genetic diversity of Chinese goats [28]. The breed with the most HWE was MG, with 13 loci showing such deviations. In this study, there were deviations between *H*_O_ and *H*_E_ in all breeds, indicating that all breeds had been disturbed to a certain extent. Deviations from HWE can arise in two situations. One is when individual populations are substructured into isolated smaller flocks within them, and the other is when populations are improperly managed by humans as a result of inbreeding [12, 31].

Inbreeding can increase the level of homozygosity within a population, thereby reducing genetic diversity [32]. The *F*_IS_ values of MG, YM, YL, GZ, and ZWL were significantly higher than those under random rearrangement (p<0.05), indicating that inbreeding occurred in these breeds. It was notable that the *F*_IS_ of the MG breed is 0.376, and it showed an extremely significant difference from the *F*_IS_ under random rearrangement (p<0.001), indicating that the level of inbreeding in this breed was extremely high. The fact that its *H*_O_ was 0.46, which was significantly lower than the *H*_E_ of 0.74, also supports this conclusion. The *F*_ST_ was the indicator for evaluating the degree of population differentiation. YL, FQ, YM, LW, CD, and MG exhibited distinct genetic differentiation from other breeds. Among them, YM and LW were from East China, while CD was from North China. The substantial genetic divergence between these breeds and those from Southwest China could be largely attributed to geographical isolation. Interestingly, despite YL, FQ, and MG also being from Southwest China, they exhibited notable genetic differentiation from other local breeds (Table 1). This phenomenon might have been linked to the unique topographical features of their habitats or the extent of their genetic exchange with external populations [33]. Moreover, the p-values for all interspecific *F*_ST_ were below the corrected p-values, all demonstrating statistical significance. This finding strongly suggested that each breed had undergone distinct genetic evolution and functions as an independent breeding unit.

The neighbor - joining tree constructed using Reynolds distance for 18 native goat breeds showed that most breeds cluster according to their geographical locations. In the studies conducted by Di et al [8], Liu et al [9], and Wei et al [28], the goat populations they investigated were also mainly clustered based on geography. YL and FQ, located on the Yunnan - Guizhou Plateau at around 25°N latitude, were grouped together. JC and GZ, also on the Yunnan - Guizhou Plateau but at 27°N latitude, form another cluster. DZ, CN, YD, and CZ from the Sichuan Basin, along with MC from the eastern Hubei Plain, were classified as one cluster. Besides being at similar latitudes and on similar terrains, they were all located along the Yangtze River. Rivers have always been closely related to cultural exchanges, which in turn promote the exchange of genetic materials among organisms [34]. Over time, their genetic materials had become relatively stable. However, the Bayesian clustering diagram in Figure 2 C showed that the bar charts of goat breeds along the Yangtze River (DZ, CN, CZ, YD, MC) contained multiple colors (in addition to the main color, green, there are also mixtures of red, blue, yellow and pink.), indicating that the exchange of genetic materials among these breeds had not ceased. These results were consistent with E et al [12] XD and LZ in the coastal area also had bar charts with diverse colors (in addition to the predominant red or blue colors, other colors also constitute a significant proportion), which was evidence of their genetic material exchanged with other breeds. In contrast, except for a few individuals, the bar chart of MG showed a very single color, indicating that MG had a relatively pure bloodline with little external interference. This was consistent with the high - level inbreeding of MG mentioned earlier.

Notably, according to the phylogenetic tree and clustering diagram, YL and FQ from Yunnan had been grouped with YM and LW from Shandong and CD from Hebei, despite the large geographical distances between them. YM, LW, and CD live in mountainous areas at an average altitude of about 400 meters under a temperate monsoon climate, while FQ and YL are in plateau areas at an altitude of about 2,000 meters under a subtropical plateau monsoon climate. We speculate that the similar climate factors such as temperature and humidity in these areas (considering that the temperature drops by 6°C for every 1,000 meters increase in altitude) may have led to similar genetic materials after long - term natural selection [35]. It is also possible that the introduction of Boer goats for the improvement of native breeds has contributed to this similarity [36].

## CONCLUSION

In our study, all 18 selected black goat breeds exhibited relatively high genetic diversity. However, MG is at risk of inbreeding depression. Additionally, we found that CZ and LZ had a high degree of cross - breeding through introduction, which to some extent masked their original genetic characteristics. Finally, we discovered that breeds from habitats with similar geographical and climatic conditions might possess relatively similar genetic materials. The main contribution of this study is a substantial analysis of the genetic diversity and genetic structure of native Chinese black goat breeds, providing a reference for the conservation and genetic improvement of these native breeds.

## Figures and Tables

**Table 1:** Sampling information of 18 black goat breeds

Breed	Breed code	Sample size	North latitude	East longitude	Geographical location	Region
Dazu black goat	DZ	44	29°42′24.48″	105°43′19.20″	Dazu, Chongqing, China	Southwest China
Chuannan black goat	CN	39	28°43′26.04″	105°4′1.20″	Yibin, Sichuan, China	Southwest China
Chuanzhong black goat	CZ	68	30°14′28.66″	105°3′12.63″	Lezhi, Sichuan, China	Southwest China
Yudong black goat	YD	29	29°19′32.52″	107°45′36.00″	Wulong, Chongqing, China	Southwest China
Baiyu black goat	BY	20	31°12′34.56″	98°49′28.20″	Baiyu, Sichuan, China	Southwest China
Jianchang black goat	JC	44	26°39′17.64″	102°14′42.00″	Huili, Sichuan, China	Southwest China
Meigu goat	MG	33	28°19′22.29″	103°7′57.50″	Meigu, Sichuan, China	Southwest China
Chengde wujiao goat	CD	35	41°25′3.34″	117°34′47.55″	Chengde, Hebei, China	North China
Laiwu black goat	LW	31	36°12′11.52″	117°39′36.00″	Laiwu, Shandong, China	East China
Yimeng black goat	YM	36	36°11′5.64″	118°10′15.60″	Yiyuan, Shandong, China	East China
Fengqing wujiao black goat	FQ	24	24°34′49.80″	99°55′42.60″	Fengqing, Yunnan, China	Southwest China
Yunling goat	YL	35	25°1′58.08″	101°32′45.60″	Chuxiong, Yunnan, China	Southwest China
Leizhou goat	LZ	37	20°54′51.12″	110°5′45.60″	Leizhou, Guangdong, China	South China
Lvliang biack goat	LL	40	37°31′6.24″	111°8′38.40″	Lvliang, Shanxi, China	North China
Macheng biack goat	MC	36	31°10′16.68″	115°0′36.00″	Macheng, Hubei, China	Central China
Xiangdong biack goat	XD	32	28°12′25.01″	113°43′9.52″	Liuyang, Hunan, China	Central China
Guizhou black goat	GZ	40	27°4′11.21″	105°9′41.62″	Bijie, Guizhou, China	Southwest China
Ziwuling black goat	ZWL	38	36°12′21.24″	107°32′15.06″	Qingyang, Gansu, China	Northwest China

**Table 2:** Primer information of sixteen microsatellites

Locus	Chromosomal location	Sequence (5′–3′) (bp)	Fragment length (bp)	Melting temperature (°C)
CSRD247 F	OAR14	GGACTTGCCAGAACTCTGCAAT	220–247	58
CSRD247 R	OAR14	CACTGTGGTTTGTATTAGTCAGG		
ILSTS005 F	BTA10	GGAAGCAATTGAAATCTATAGCC	172–218	55
ILSTS005 R	BTA10	TGTTCTGTGAGTTTGTAAGC		
INRA063 F	CHI18	GACCACAAAGGGATTTGCACAAGC	164–186	58
INRA063 R	CHI18	AAACCACAGAAATGCTTGGAAG		
INRABERN185 F	CHI18	CAATCTTGCTCCCACTATGC	261–289	55
INRABERN185 R	CHI18	CTCCTAAAACACTCCCACACTA		
MAF065 F	OAR15	AAAGGCCAGAGTATGCAATTAGGAG	116–158	58
MAF066 R	OAR15	CCACTCCTCCTGAGAATATAACATG		
MAF209 F	CHI17	GATCACAAAAAGTTGGATACAACCGTG	100–104	55
MAF209 R	CHI17	TCATGCACTTAAGTATGTAGGATGCTG		
OarAE54 F	OAR25	TACTAAAGAAACATGAAGCTCCCA	115–138	58
OarAE54 R	OAR25	GGAAACATTTATTCTTATTCCTCAGTG		
OarFCB20 F	OAR2	GGAAAACCCCCATATATACCTATAC	93–112	58
OarFCB20 R	OAR2	AAATGTGTTTAAGATTCCATACATGTG		
SPS113 F	BTA10	CCTCCACACAGGCTTCTCTGACTT	134–158	58
SPS113 R	BTA10	CCTAACTTGCTTGAGTTATTGCCC		
SRCRSP15 F	Unknown	CTTTACTTCTGACATGGTATTTCC	172–198	55
SRCRSP15 R	Unknown	TGCCACTCAATTTAGCAAGC		
SRCRSP23 F	Unknown	TGAACGGGTAAAGATGTG	81–119	58
SRCRSP23 R	Unknown	TGTTTTTAATGGCTGAGTAG		
SRCRSP5 F	CHI21	GGACTCTACCAACTGAGCTACAAG	156–178	55
SRCRSP5 R	CHI21	TGAAATGAAGCTAAAGCAATGC		
SRCRSP7 F	CHI6	TCTCAGCACCTTAATTGCTCT	117–131	55
SRCRSP7 R	CHI6	GGTCAACACTCCAATGGTGAG		
SRCRSP8 F	Unknown	TGCGGTCTGGTTCTGATTTCAC	215–255	55
SRCRSP8 R	Unknown	GTTTCTTCCTGCATGAGAAAGTCGATGCTTAG		
SRCRSP9 F	OAR17	AGAGGATCTGGAAATGGAATC	99–135	58
SRCRSP9 R	OAR17	GCACTCTTTTCAGCCCTAATG		
TCRVB6 F	BTA10	GAGTCCTCAGCAAGCAGGTC	217–255	55
TCRVB6 R	BTA10	CCAGGAATTGGATCACACCT		

**Table 3:** Estimated values of genetic diversity indices at 16 loci for 18 black goat breeds

Loci	NA	*H*_O_	*H*_E_	PIC
CSRD247	26	0.68	0.73	0.69
ILSTS005	12	0.51	0.54	0.47
INRA063	15	0.71	0.72	0.67
INRABERN185	22	0.35	0.38	0.34
MAF065	23	0.73	0.77	0.73
MAF209	7	0.31	0.36	0.29
OarAE54	25	0.67	0.73	0.69
OarFCB20	20	0.63	0.68	0.63
SPS113	23	0.69	0.69	0.64
SRCRSP5	21	0.67	0.71	0.66
SRCRSP7	13	0.22	0.39	0.35
SRCRSP8	24	0.74	0.8	0.76
SRCRSP9	25	0.66	0.69	0.65
SRCRSP15	12	0.48	0.49	0.44
SRCRSP23	30	0.64	0.74	0.7
TCRVB6	30	0.7	0.72	0.68
Mean	20.5	0.59	0.63	0.59

NA, number of alleles; *H*_O_, observed heterozygosity; *H*_E_, expected heterozygosity; PIC, polymorphism information content.

**Table 4:** Diversity parameters in 18 Chinese black goat breeds

Breed	Breed code	N	Allelic diversity			Genetic diversity					

			TNA	NEA±SD	MNA±SD	AR±SD	NPA	*H*_O_±SD	*H*_E_±SD	HWE	*F*_IS_
Dazu	DZ	44	85	2.81±1.24	5.31±2.30	4.41±1.62	0	0.586±0.1	0.59±0.18	0	0.001
Chuannan	CN	39	90	2.99±1.26	5.63±2.00	4.64±1.52	0	0.58±0.20	0.60±0.21	4	0.034
Chuanzhong	CZ	68	104	3.24±1.58	6.50±2.42	5.18±1.78	0	0.60±0.24	0.60±0.21	2	−0.003
Yudong	YD	29	96	3.13±1.08	6.00±2.25	5.15±1.70	1	0.63±0.21	0.64±0.19	1	0.007
Baiyu	BY	20	89	2.84±1.13	5.56±2.19	5.07±1.89	1	0.56±0.19	0.60±0.20	0	0.066
Jianchang	JC	44	76	2.69±0.94	4.75±1.53	4.02±1.17	0	0.57±0.16	0.59±0.15	1	0.033
Meigu	MG	33	153	4.11±1.57	9.56±4.19	7.26±2.45	51	0.46±0.21	0.74±0.10	13	0.376^***^
Chengde	CD	35	95	2.91±1.42	5.94±2.38	4.96±1.85	3	0.49±0.26	0.56±0.25	6	0.122
Laiwu	LW	31	119	4.18±1.70	7.44±2.10	6.53±1.78	10	0.68±0.19	0.73±0.13	3	0.070
Yimeng	YM	36	141	3.90±2.10	8.81±3.58	6.72±2.63	13	0.59±0.22	0.65±0.23	5	0.100^**^
Fengqing	FQ	24	79	2.65±0.90	4.93±1.88	4.39±1.52	5	0.57±0.22	0.59±0.16	4	0.033
Yunling	YL	35	107	3.50±1.60	6.69±3.26	5.47±2.27	7	0.52±0.20	0.66±0.18	9	0.207^**^
Leizhou	LZ	37	93	2.95±1.30	5.81±2.22	4.72±1.51	1	0.58±0.24	0.59±0.22	3	0.023
Lvliang	LL	40	122	4.07±1.61	7.63±2.75	6.22±1.98	7	0.68±0.22	0.70±0.20	1	0.022
Macheng	MC	36	98	3.33±1.54	6.13±2.16	5.20±1.66	0	0.61±0.20	0.64±0.17	2	0.045
Xiangdong	XD	32	78	2.76±0.90	4.86±1.89	4.22±1.54	0	0.58±0.24	0.59±0.19	2	0.022
Guizhou	GZ	40	99	3.29±1.18	6.19±2.56	5.16±1.77	0	0.63±0.16	0.66±0.13	3	0.049^*^
Ziwuling	ZWL	38	113	4.06±1.73	7.06±2.11	5.99±1.74	4	0.67±0.22	0.69±0.19	2	0.040^*^
Mean			102.06	3.30±0.54	6.38±1.32	5.30±0.91	5.72	0.59±0.06	0.63±0.05	3.39	

The p of *F*_IS_ were obtained based on 1,023 random permutations.

* indicates p<0.05,

** indicates p<0.01, and

*** indicates p<0.001.

N, number of samples; TNA, total number of alleles; NEA, number of effective alleles; SD, standard deviation; MNA, mean number of alleles; AR, allelic richness; NPA, number of private alleles; *H*_O_, observed heterozygosity; *H*_E_, expected heterozygosity; HWE, the number of loci deviating from Hardy-Weinberg equilibrium; *F*_IS_, inbreeding coefficient.

**Table 5:** Pairwise differences in population averages (Slatkins linearized *F*_ST_) using microsatellite markers

	DZ	CN	CZ	YD	BY	JC	MG	CD	LW	YM	FQ	YL	LZ	LL	MC	XD	GZ	ZWL
DZ	0																	
CN	0.0346^*^	0																
CZ	0.0289^*^	0.0195^*^	0															
YD	0.0352^*^	0.0247^*^	0.0191^*^	0														
BY	0.0953^*^	0.1141^*^	0.096^*^	0.0797^*^	0													
JC	0.0710^*^	0.0812^*^	0.0813^*^	0.0711^*^	0.0881^*^	0												
MG	0.2642^*^	0.2659^*^	0.2684^*^	0.2407^*^	0.2500^*^	0.2501^*^	0											
CD	0.3390^*^	0.3575^*^	0.3394^*^	0.3303^*^	0.3521^*^	0.3397^*^	0.2188^*^	0										
LW	0.2648^*^	0.2831^*^	0.2620^*^	0.2533^*^	0.2850^*^	0.2707^*^	0.1233^*^	0.118^*^	0									
YM	0.2936^*^	0.3091^*^	0.2999^*^	0.2846^*^	0.2966^*^	0.2942^*^	0.1708^*^	0.042^*^	0.0498^*^	0								
FQ	0.3258^*^	0.3291^*^	0.3259^*^	0.3074^*^	0.3183^*^	0.2999^*^	0.162^*^	0.2272^*^	0.1744^*^	0.1855^*^	0							
YL	0.2944^*^	0.2994^*^	0.2954^*^	0.2786^*^	0.2902^*^	0.2677^*^	0.1649^*^	0.1529^*^	0.0832^*^	0.1157^*^	0.1488^*^	0						
LZ	0.0854^*^	0.0752^*^	0.072^*^	0.0596^*^	0.1184^*^	0.1301^*^	0.2658^*^	0.34^*^	0.2678^*^	0.2934^*^	0.3243^*^	0.2969^*^	0					
LL	0.0813^*^	0.0772^*^	0.0675^*^	0.0624^*^	0.062^*^	0.0998^*^	0.2049^*^	0.2792^*^	0.2072^*^	0.2352^*^	0.2811^*^	0.2376^*^	0.0865^*^	0				
MC	0.0304^*^	0.0342^*^	0.0144^*^	0.0254^*^	0.0875^*^	0.0697^*^	0.2367^*^	0.3176^*^	0.2370^*^	0.2717^*^	0.3007^*^	0.2684^*^	0.054^*^	0.0523^*^	0			
XD	0.0736^*^	0.0736^*^	0.0693^*^	0.0647^*^	0.1093^*^	0.1151^*^	0.2696^*^	0.3259^*^	0.2729^*^	0.2858^*^	0.3232^*^	0.296^*^	0.0795^*^	0.0677^*^	0.0562^*^	0		
GZ	0.0405^*^	0.0561^*^	0.042^*^	0.0335^*^	0.0586^*^	0.0324^*^	0.2248^*^	0.3026^*^	0.2262^*^	0.2628^*^	0.2787^*^	0.2454^*^	0.0862^*^	0.054^*^	0.0309^*^	0.0725^*^	0	
ZWL	0.0777^*^	0.0818^*^	0.0727^*^	0.0565^*^	0.0501^*^	0.0929^*^	0.2006^*^	0.269^*^	0.2013^*^	0.2255^*^	0.2748^*^	0.2388^*^	0.0683^*^	0.0136^*^	0.0499^*^	0.055^*^	0.0524^*^	0

* Indicates that the p-value is less than the corrected p-value (using the Bonferroni correction with a significance level of 0.05. A total of 110 operations were carried out when calculating the p-value of *F*_ST_. The corrected p-value is 0.05/110 = 0.00045).

DZ, Dazu; CN, Chuannan; CZ, Chuanzhong; YD, Yudong; BY, Baiyu; JC, Jianchang; MG, Meigu; CD, Chengde; LW, Laiwu; YM, Yimeng; FQ, Fengqing; YL, Yunling; LZ, Leizhou; LL, Lvliang; MC, Macheng; XD, Xiangdong; GZ, Guizhou; ZWL, Ziwuling. (These are all breed names of Chinese native black goats.)
